# Insights Into Arsenite and Arsenate Uptake Pathways Using a Whole Cell Biosensor

**DOI:** 10.3389/fmicb.2018.02310

**Published:** 2018-10-02

**Authors:** Martin P. Pothier, Aaron J. Hinz, Alexandre J. Poulain

**Affiliations:** Department of Biology, University of Ottawa, Ottawa, ON, Canada

**Keywords:** arsenic speciation, arsenic uptake, arsenite, arsenate, whole cell biosensor, Giant Mine, water quality

## Abstract

Despite its high toxicity and widespread occurrence in many parts of the world, arsenic (As) concentrations in decentralized water supplies such as domestic wells remain often unquantified. One limitation to effective monitoring is the high cost and lack of portability of current arsenic speciation techniques. Here, we present an arsenic biosensor assay capable of quantifying and determining the bioavailable fraction of arsenic species at environmentally relevant concentrations. First, we found that inorganic phosphate, a buffering agent and nutrient commonly found in most bioassay exposure media, was in fact limiting As(V) uptake, possibly explaining the variability in As(V) detection reported so far. Second, we show that the nature of the carbon source used in the bioassay differentially affects the response of the biosensor to As(III). Finally, our data support the existence of non-specific reduction pathways (non-*ars* encoded) that are responsible for the reduction of As(V) to As(III), allowing its detection by the biosensor. To validate our laboratory approach using field samples, we performed As(III) and As(V) standard additions on natural water samples collected from 17 lakes surrounding Giant Mine in Yellowknife (NWT), Canada. We found that legacy arsenic contamination in these lake water samples was accurately quantified by the biosensor. Interestingly, bioavailability of freshly added standards showed signs of matrix interference, indicative of dynamic interactions between As(III), As(V) and environmental constituents that have yet to be identified. Our results point toward dissolved organic carbon as possibly controlling these interactions, thus altering As bioavailability.

## Introduction

Arsenic contamination of drinking water poses a significant human health risk worldwide (Andy et al., 2017). The occurrence of arsenic in lakes and ground water is closely linked with geologic sources (Bhattacharya et al., 2007) and can be exacerbated by anthropogenic activities (Riveros et al., 2001). Chronic ingestion of geogenic arsenic from contaminated water increases the risk of several cancers and multisystem diseases (Chan and Huff, 1997; Kitchin, 2001; IARC, 2013). In efforts to reduce arsenic poisoning, the WHO has set the maximum allowable concentration of arsenic in drinking water to 10 μg/L or 133 nM (WHO, 2004).

Despite its high toxicity and widespread occurrence, in many parts of the world (Chen and Wang, 1990; George et al., 2014; Flanagan et al., 2016), arsenic concentrations in decentralized water supplies such as domestic wells remain often unquantified. One barrier to effective monitoring is the high cost and lack of portability of analytical techniques (Melamed, 2005). Commonly used methods of analyzing arsenic in environmental samples involve separation of chemical species by high-performance liquid chromatography (HPLC). Methods for quantification include inductively coupled plasma mass spectrometry (ICP-MS) and atomic fluorescence spectroscopy (AFS). While these techniques reliably provide accurate quantification of arsenic, purchasing and operation costs of these instruments is prohibitive for large-scale monitoring (Melamed, 2005).

A complementary approach to arsenic quantification involves the use of microbial biosensors (Corbisier et al., 1993; Trang et al., 2005; Cortés-Salazar et al., 2013; Truffer et al., 2014) that produce quantifiable signals in response to arsenic exposure. Most arsenic biosensors are based on regulation of reporter genes by an arsenic sensing transcriptional repressor (ArsR) (Wu and Rosen, 1993). Output signals of reporter genes include light emission (e.g., luciferase) (Tauriainen et al., 1997; Trang et al., 2005; Zhang et al., 2009) and fluorescence (e.g., green fluorescent protein) (Stocker et al., 2003; Chen et al., 2014). Because production of the signal requires uptake of arsenic by live cells, biosensors quantify, to some extent, the fraction of arsenic that is available to microbes for transformations. This bioavailable fraction cannot be determined by other analytical techniques like HPLC-ICP-MS or chemical test kits. Moreover, biosensors hold promise for quantifying arsenic in remote locations (Trang et al., 2005; Date et al., 2007; Siegfried et al., 2012; Cortés-Salazar et al., 2013; Roggo and van der Meer, 2017), where the use of other analytical methods is impractical.

One potential limitation of arsenic biosensors is their inability to distinguish between different chemical species of arsenic. Arsenic in contaminated drinking water is typically inorganic and can exist in two redox states [As(III) and As(V)], which differ in their mobility, bioavailability, and toxicity (Reeder et al., 2006; Chang et al., 2008). Arsenic speciation is governed by physico-chemical conditions of the environment, particularly pH and redox potential (Lu and Zhu, 2011). In anoxic groundwater, As is generally present as As(III), often released by dissolution of As-containing minerals (Valenzuela, 2000). Once in contact with oxic surface water, As(III) is oxidized to As(V) (Valenzuela, 2000). Microbes also play a key role in controlling arsenic speciation. The genetic determinants involved in As metabolism and cycling are thought to be ancient (Sforna et al., 2014) and diverse. Indeed, microbes have been shown to oxidize As(III) [e.g., via the *aio* operon, *aso* or *aox* gene clusters (Santini and vanden Hoven, 2004; Silver and Phung, 2005; Cai et al., 2009)], reduce As(V) for catabolic or detoxification purposes [e.g., via the *ars* and *arr* operons (Wu and Rosen, 1993; Saltikov and Newman, 2003)], as well as catalyze the formation of several organoarsenic species [e.g., via *ars*M (Bentley and Chasteen, 2002; Ye et al., 2012)].

Whereas ArsR-based sensors (i.e., derived from the *ars* operon) have been routinely used to detect inorganic arsenic [As(III) and As(V)] (Stocker et al., 2003; Date et al., 2007; Siegfried et al., 2012; Sharma et al., 2013), output signal is often weaker in response to As(V) (Stocker et al., 2003; Sharma et al., 2013). As(V) detection is likely to require a reduction step to As(III) before binding to ArsR, because As(III) is the only arsenic species known to interact with ArsR (Wu and Rosen, 1993; Shen et al., 2013). Thus, the weaker signal has been presumed to result from delayed or inefficient reduction of As(V) by the arsenate specific reductase (ArsC) (Stocker et al., 2003; Sharma et al., 2013). Alternatively, the inability to reliability detect As(V) may stem from differentially reduced bioavailability of As(V) in biosensor exposure assays. These limitations can lead to lower sensitivity and to the underestimation of arsenic concentrations in environmental samples.

We hypothesized that variable responses of arsenic biosensors to As may stem from heterogeneity of exposure conditions that interfere with the bioavailability of inorganic arsenic species. For instance, As(V) complexes ($H_{2}AsO_{4}^{-}$) have similar size, charge and structure to inorganic phosphate (Pi) complexes ($H_{2}PO_{4}^{-}$), and studies have found that Pi transporters are responsible for As(V) uptake (Rosenberg et al., 1977; Willsky and Malamy, 1980). In contrast, As(III) was found to be transported passively via glycerol uptake facilitator channels (GlpF in *Escherichia coli*) (Sanders et al., 1997; Rosen and Tamás, 2010). Therefore, we predicted that speciation of arsenic can be determined by manipulating media constituents that differentially alter arsenic transport of both inorganic species.

In this study, we evaluated the ability of an *E. coli* ArsR-based biosensor to quantify inorganic As species over a concentration range encompassing the World Health Organization’s guideline for arsenic in drinking water. We showed how alteration of media constituents can impact the bioavailability of arsenic species. We identified conditions permissive for the detection of both As(III) and As(V), conditions selective for detection of As(III) alone, and explored mechanisms responsible for species specific detection. We also examined the effects of chemical constituents commonly found in environmental samples that may differentially affect the bioavailability of arsenic species. Finally, we validated our proposed methodology by estimating total inorganic arsenic concentrations in natural lake water samples that cover a wide-range of chemical compositions.

## Materials and Methods

### Arsenic Biosensor Construction

Our biosensing construct was inspired by the design of Stocker et al. (2003), for which two ArsR binding sites were shown to provide optimal detection while minimizing noise (**Supplementary Figure S4**). The sensing-reporting sequence (ArsRBS2-mCherry) was constructed by custom gene synthesis (Integrated DNA Technologies) and cloned into the XmaI and XbaI restriction sites of the high copy pUCP19 shuttle vector (Schweizer, 1991; Blattner et al., 1997) upstream of the sequence encoding mCherry (Shaner et al., 2004; Shaner et al., 2005). The reporter plasmid was transformed into *E. coli* NEB10-beta (New England BioLabs) – a level 1, non-pathogenic, non-regulated host. Other than the chromosomal *ars* operon, *E. coli* NEB10-beta does not carry any other known genetic determinants annotated as involved in arsenic specific transformations.

### Construction of the *arsC* Deletion Mutant

The chromosomal arsenate-reductase gene (*arsC*) was deleted from the *E. coli* NEB10-beta genome using lambda Red recombination (Datsenko and Wanner, 2000). The Δ*arsC* mutant from the Keio collection (Strain JW3470), in which the coding sequence is replaced by a kanamycin resistance gene flanked by FLP recognition target (FRT) sequences (Baba et al., 2006), was obtained from the Coli Genetic Stock Center. The FRT-kan cassette and flanking *E. coli* genomic sequences were PCR-amplified from chromosomal DNA isolated from the Δ*arsC* mutant using primers F-Delta-arsC (5′-GTGCTGTTTGTGACGCTGG-3′) and R-Delta-arsC (5′-GCGCACTTTTCTAACAACCTGT-3′). Purified PCR products were transformed into electrocompetent NEB10-beta cells induced to express recombination functions from the Red helper plasmid pKD46, as previously described (Datsenko and Wanner, 2000). Recombinants were selected by plating on LB with 30 μg/mL kanamycin and cured of the temperature-sensitive pKD46 plasmid by incubation at 37°C. Replacement of the *arsC* gene by the FRT-kan cassette was verified by colony PCR, and plasmid curing was confirmed by testing for ampicillin sensitivity. The kanamycin-resistance gene was subsequently excised by FLP recombination with the plasmid pCP20, as previously described (Datsenko and Wanner, 2000), followed by plasmid curing, yielding putative Δ*arsC*::FRT mutants susceptible to both kanamycin and ampicillin. Colony PCR with the *arsC* deletion primers yielded a shorter amplicon for the putative Δ*arsC*::FRT mutants in comparison to the wild type, confirming the deletion of *arsC* sequences. The minimal inhibitory concentrations (MICs) of As(III) and As(V) were determined for both the wild-type and Δ*arsC* strains (**Supplementary Figure S1**). Declines in all mutant fitness measurements (i.e., growth rate, lag time and yield) occurred at an As(V) concentration 10-fold lower than that of the wild type (50 μM vs. 500 μM). In contrast, decline in fitness measurements for As(III) were similar for both strains (500 μM). Together, this data confirms that the removal of arsenate reductase from the host genome increases sensitivity to As(V).

### Media and Reagents

Ultrapure water was used in all media and reagents. The water purification process involved filtration with the Milli-Q system (Millipore), autoclaving and re-filtration at 0.2 μm. Cells hosting the biosensing construct were plated on LB agar (10 g tryptone, 5 g yeast extract, 5 g NaCl and 15 g agar per liter) with 120 μg/mL ampicillin. Before each assay, cells were pre-grown overnight in MGP (Mops Glycero-Phosphate) growth medium, a defined minimal medium free of inorganic phosphates (**Supplementary Table S1**). Constituents include 20 mM MOPS as a buffering agent, 1 mM β-glycerophosphate (BGP) as a phosphate source and 30 mM glycerol as the primary carbon source. The growth medium was supplemented with trace elements and amino acids (L-Leucine, L-Isoleucine and Valine) as *E. coli* NEB 10-beta is an auxotrophic strain [Δ(ara-leu)] for these compounds (**Supplementary Table S1**).

The non-selective arsenic exposure medium (**Supplementary Table S1**) is a modified MGP growth medium with no trace elements, reduced [Mg^2+^] (20 μM) and reduced [glycerol] (5 mM). Discriminating between As(III) and As(V) is possible using MIP (MOPS Inorganic Phosphate) exposure medium, which is an MGP exposure medium supplemented with 10 mM inorganic phosphate (**Supplementary Table S1**). In order to test for the role of different carbon sources on As(III) uptake, the MGP exposure medium required the following substitutions: 1 mM BGP with 1 mM Pi, and 5 mM glycerol with 5 mM glucose.

Biosensor As standards consisted of sodium (meta)arsenite (NaAsO_2_; Cat#S7400-100G) and sodium arsenate dibasic heptahydrate (Na_2_HAsO_4_ ⋅ 7H_2_O; Cat#A6756-100G), purchased from Sigma-Aldrich. Arsenic salts were dissolved in ultrapure water and acidified with HNO_3_ to a final concentration of 10 mM As and 0.1% HNO_3_. Arsenic standards were kept up to 4 weeks in 15 mL polypropylene tubes at 4°C in the absence of light. Arsenic species in our standard solutions were periodically verified using HPLC-ICP-MS and deemed stable for 1 month. Working standards of 10 μM were prepared daily before exposure to sensor cells.

Reagents used in ICP-MS quantification and speciation were of analytical grade and used without further purification. Arsenic standards and other reagents were purchased from Sigma-Aldrich and Spex Certiprep. Stock solutions of 1000 mg/L of arsenite (Spex Certiprep, Cat#SPEC AS3M), and arsenate (Spex Certiprep, Cat#SPEC-AS5M) with a certified value of arsenic traceable to a NIST Standard Reference materials were purchased from Spex Certiprep. A 10 mM solution of ammonium phosphate dibasic was prepared by dissolving ammonium phosphate dibasic (Sigma, cat#379980-100G) in ultrapure water and pH adjusted to 8.25 with 28% Ammonium hydroxide solution (Sigma, Cat#: 338818-100ML). Mobile phase was filtered through a 0.45 μm filter before use.

### Arsenic Exposure and Quantification by the Biosensor

Growth was initiated by transferring a single colony into MGP growth medium supplemented with 1% LB and 100 μg/mL ampicillin in round-bottom culture tubes with a 1 cm path length. Cultures were incubated aerobically overnight at 200 RPM and 37°C until they reached an OD_600_ (optical density at 600 nm) range of 1.1–1.2. Before exposure to arsenic, cells were statically incubated at room temperature for 3 h and diluted to an OD_600_ of 1.050 using MGP growth medium. This culture was used as the inoculum in subsequent assays.

Unless otherwise noted, all arsenic exposure assays include adding the following components in the following order: (1) exposure medium, (2) reagents, (3) water, and (4) cells. Medium to inoculum ratio was consistent in all treatments by concentrating exposure media twofold. Step 1 involved the transfer of 800 μL of 2X concentrated MGP or MIP to a 7 mL borosilicate scintillation vial. Step 2 involved the addition of reagents such as arsenic and/or salts to the vials. Reagent working solutions were adjusted to allow a final dilution that does not exceed a total of 1 mL. During step 3, ultrapure water was used to adjust the final volume of reagents to 1 mL. Here, reagents and media constituents were incubated in the vials for 15 min at room temperature with periodical orbital shaking by hand. Finally, 200 μL of unwashed biosensor cell cultures were added to all treatments. Vials were incubated at 37°C for 20 min at 200 RPM. In all assays, plating consisted of technical triplicates by adding 200 μL of each treatment in three separate wells of a Corning 96 well black plate with a non-binding surface and a clear bottom. Using a Tecan Infinite F200 Pro plate reader, fluorescence and OD_600_ of a 0.6 cm path length was measured from the top of the plate on kinetic cycles of 10 min for 22 h at 37°C and 25 flashes per read. We used an optically clear lid to minimize evaporation throughout the incubation. Fluorescence intensity was analyzed using 560/20× nm excitation and 620/10× nm emission filters with gain manually set to 100.

### Speciation Analysis of Cell and Supernatant Fractions

Using the previously described exposure protocol, 5 mL cultures of both wild-type and Δ*arsC* mutant strains were incubated at 37°C with 32 μM As(V) for 3 h in MGP exposure media. To determine the fraction and speciation of arsenic inside the cells, cultures pre- and post-incubation were centrifuged at 15 000 RPM for 3 min. Pelleted cells were washed twice using MGP exposure media rather than ultrapure water to avoid cell lysis during washes. The use of preservation agents such as acids were also avoided for this same reason. Rather, pellets were separated from supernatant, placed on ice and analyzed on the same day in biological triplicates using HPLC-ICP-MS. No-cell controls underwent identical methodology including centrifugation and icing steps. As(III) was not detected in the controls, indicating that As(V) was not reduced by constituents in the growth medium nor from the steps used to fractionate cells from supernatant. Our mass balance for all biological replicates indicated that we retrieved ca. 100% of the As(V) added.

### Analysis of Glucose Concentrations in Culture Supernatant

Methods involving the monitoring of glucose consumption over time required an increase of the final volume to 12 mL. For each treatment, 200 μL was subsampled and plated for kinetic RFU and OD_600_ quantification. The remaining volume was incubated at 37°C and used for glucose consumption monitoring, where a 1 mL subsample was extracted/filter sterilized (0.2 μm) every 1.5 h for 10.5 h. Samples were frozen and stored for 1 week before analysis by Evaporative Light Scattering Detector (ELSD) coupled to High Pressure Liquid Chromatography (HPLC). Glucose concentration could only be accurately quantified from the initial 5 mM concentration until it reached a concentration of 2 mM. We found that one of the constituents common to both glucose and glycerol media led to an increase in the baseline at the 13.5-min retention time making quantification of glucose not possible beyond this point.

### Collection and Chemical Analysis of Environmental Lake Samples

In September 2017, water samples were collected by helicopter from the middle of 17 lakes near Yellowknife (Northwest Territories, Canada). At each site, two samples were collected where one was dedicated for water chemistry and the other for arsenic analysis. Water chemistry data was provided by Taiga Environmental Laboratory, a full service analytical laboratory located in Yellowknife, accredited by the Canadian Association for Laboratory Accreditation (CALA) to ISO/IEC 17025 standards. Unfortunately, we were not able to obtain Pi concentrations for these lakes because of major interferences between PO_4_^3-^ and AsO_4_^3-^ analyses. Samples dedicated for arsenic speciation analysis were collected using HDPE (high density polyethylene) containers, kept in the dark and chilled before cross-analysis by the biosensor and HPLC-ICP-MS in Ottawa, ON, Canada. Our study prioritized the preservation of natural matrix constituents (e.g., organic matter, colloids) rather than the preservation of arsenic species. Therefore, we refrained from filtering and acidifying these lake samples that may have affected the bioavailability of As or the viability of the cells once added to the assay. HPLC-ICP-MS speciation analysis revealed that although a small fraction of the arsenic was in the form of arsenobetaine, over 99% of the arsenic was in the oxidized inorganic form As(V), as expected from unpreserved samples. Cross-analysis between the biosensor and the HPLC-ICP-MS analyses were conducted on the same day.

Biosensor analysis of arsenic concentration for 9 of 17 lakes was above the linear range of the calibration curve and required a 20× dilution before quantification (100 μL of lake water, 900 μL ultrapure water, 800 μL 2x MGP and 200 μL biosensor culture). A 2x dilution was used for the 8 remaining lakes (1000 μL lake water, 800 μL of 2x MGP and 200 μL biosensor culture). For the arsenic standard addition protocol (**Supplementary Figure S2**), 10 μM As working solutions were prepared in 2x MGP exposure media rather than ultrapure water. This modification to the arsenic exposure protocol ensured that lake water would not be diluted by the spiked arsenic.

## Results and Discussion

### Arsenic Detection and Speciation Determination by a Fluorescent Biosensor

**FIGURE 1:**
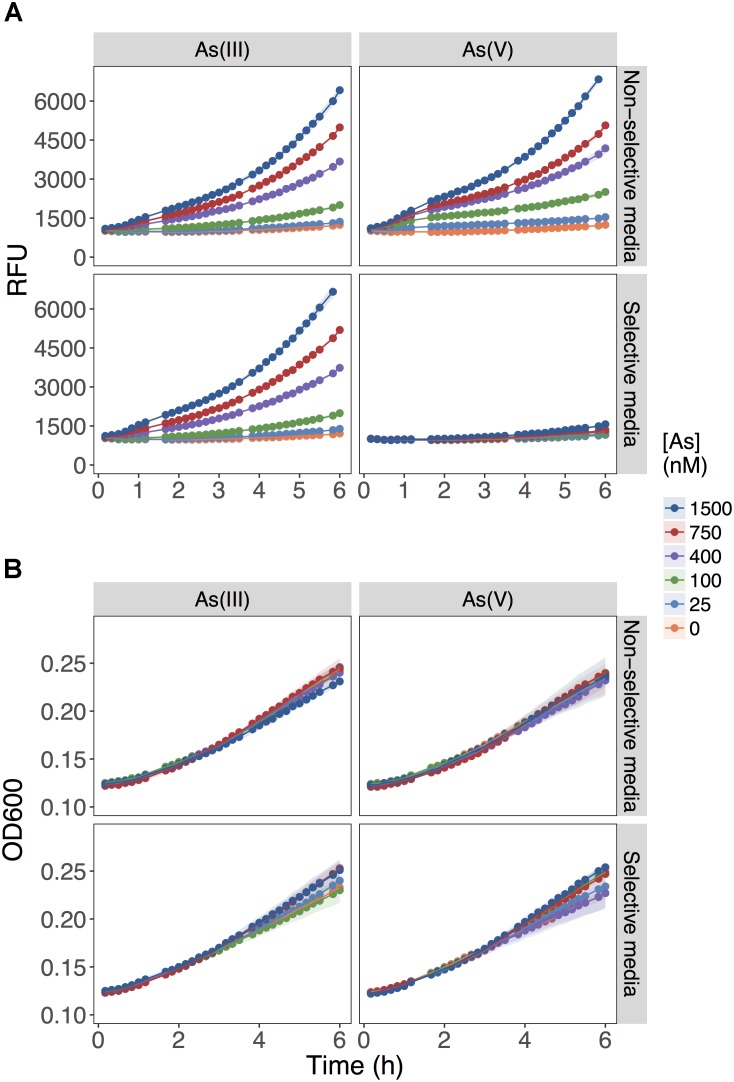
Role of inorganic phosphate addition in detection of As(III) and As(V). **(A)** Biosensor was exposed to the indicated concentrations of As(III) or As(V) in MGP media lacking inorganic phosphate (non-selective media) or MIP media containing 10 mM inorganic phosphate (selective media). The output signal of the biosensor is presented in relative fluorescence units (RFU) during the first 6 h of growth immediately following exposure to arsenic. **(B)** The optical densities of biosensor cultures at 600 nm (OD_600_) are shown, indicating similar growth rates for the cultures in both exposure media.

The biosensor detected equally well As(III) and As(V) when each As species was provided independently as the sole source of As in the non-selective medium, MGP (**Figure 1A**; upper panels). The fluorescent signal output was linearly proportional to the concentration of both arsenic species from 25 to 800 nM. As predicted, the presence of inorganic phosphate (10 mM) in the selective medium (MIP) prevented the detection of As(V) up to [As(V)] = 1500 nM, while minimally affecting As(III) detection (**Figure 1A**; lower panels). Under all conditions tested, growth was not affected (**Figure 1B**), indicating that substitution of inorganic phosphate for BGP did not limit growth rate nor yield.

**FIGURE 2:**
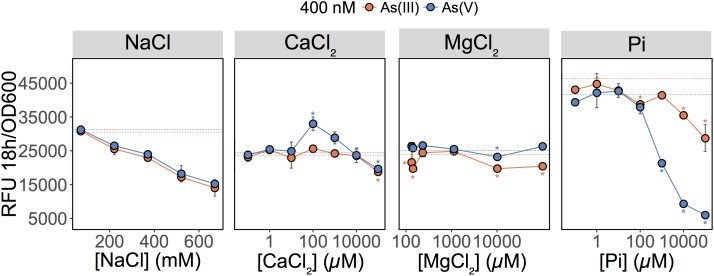
Effect of major ions on arsenic species detection. Detection of As(III) and As(V) in MGP exposure medium supplemented with NaCl, inorganic phosphate (Pi), CaCl_2_, or MgCl_2_. Pi was supplemented as a mixture of sodium/potassium phosphate salts (**Supplementary Table S1**). The arsenic concentration was 400 nM in each treatment. The mean and standard deviation of RFU per OD_600_ after 18 h of growth is presented for triplicate samples. Left panel, no significant differences were observed between linear regressions of As(III) and As(V) output signal over the NaCl concentration gradient (*p* = 0.07). In conjunction with ANOVAs, TukeyHSD was used for calcium, magnesium, and phosphate treatments. Stars indicate a statistically significant difference in output signals when compared to the control (dotted line; no added ions).

These results showed that As(V) bioavailability, and thus detection, was dependent on Pi concentrations. Previous work in *E. coli* (Willsky and Malamy, 1980) and plants (Abedin et al., 2002; Meharg and Hartley-Whitaker, 2002) have established that Pi and As(V) compete for cellular uptake, but so far, this knowledge had not been fully exploited in biosensor applications. As previously suggested, the inhibition of As(V) uptake at high Pi concentrations could arise from (i) competition between Pi and As(V) at Pi transporter sites, or (ii) repression of the high-affinity phosphate transporter (Pst), which occurs at Pi concentrations exceeding 20 μM (Rao and Torriani, 1990). We also show that inclusion of excess Pi (>100 μM) in the exposure media selectively blocked detection of As(V) with minimal effect on the detection of As(III) (*p* = 0.04) (**Figure 2**).

We were also interested in investigating the effects of other major freshwater ions such as Ca^2+^, Mg^2+^, and Na^+^ on the detection of As(III) and As(V) by the biosensor (**Figure 2**; center left and right panels). We altered the ionic strength of the medium by increasing sodium chloride (NaCl) concentrations to those representative of seawater (600 mM) (**Figure 2**; center left panel). Interestingly, as NaCl concentrations increased, the fluorescent signal was equally reduced for both the As(III) and As(V) (*p* = 0.073). Here, signal reduction was associated with the inhibitory effect of NaCl on growth rather than a change in As species bioavailability (data not shown). Additions of calcium chloride (CaCl_2_) and magnesium chloride (MgCl_2_) in MGP exposure medium had little effect on the detection of As; a slight enhancement in bioavailability was observed for [Ca^2+^] = 100 μM (**Figure 2**).

### Quantifying Arsenic Species in Samples Containing Both As(III) and As(V)

**FIGURE 3:**
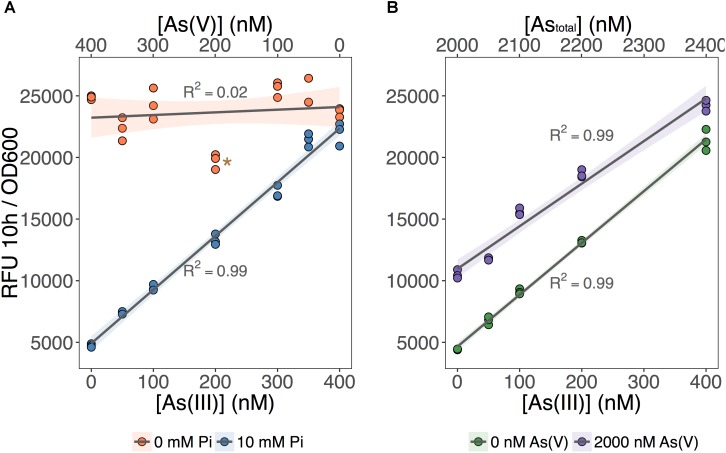
As(III) can be detected independent of As(V) concentration. **(A)** The biosensor was exposed to samples that contained 400 nM total inorganic arsenic and variable input concentrations of As(V) and As(III), indicated on the upper and lower *x*-axes. MGP (0 mM Pi) and MIP (10 mM Pi) exposure media were used for detection of total inorganic arsenic and As(III), respectively. **(B)** As(III) was detected using MIP exposure media in samples containing 0 nM or 2000 nM As(V). Input concentrations of As(III) are presented on the *x*-axis. On both plots, triplicate RFU per OD_600_ treatment points are presented rather than means with error bars. The star indicates a statistically significant difference in output signals when compared to the regression analysis.

We tested the ability of our biosensor to selectively detect As(III) over a wide range of As(V) concentrations (**Figure 3**). We first set total As level at 400 nM while varying the relative concentrations of each species (**Figure 3A**; [As] indicated on the *X*-axes). We showed that MGP exposure media can accurately quantify total inorganic arsenic concentration (orange circles: no Pi added) irrespective of the relative proportion of each arsenic species. In contrast, fluorescence in the As(III)-selective exposure media (blue circles: 10 mM Pi) was proportional to the As(III) input concentration, indicating that [As(III)] was accurately quantified irrespective of background [As(V)].

Next, we tested the effect of adding a large excess of As(V) (2 μM) on the detection of As(III) using the As(III)-selective exposure medium (MIP). The output signal was proportional to the concentration of As(III) in both exposure conditions, regardless of the presence of As(V) (**Figure 3B**). We noted, however, that the baseline output signal (intercept) for the detection of As(III) in the presence of 2 μM As(V) treatments was significantly higher. This increase in baseline output signal corresponds to an overestimation of ∼100 nM across each [As(III)] tested. We attribute this baseline shift to the presence of a threshold, where 10 mM Pi is effective at excluding low background [As(V)]. However, once this threshold is exceeded, we estimate that 10–15% of background As(V) can enter the cell and increase signal output.

### As(V) Detection Involves an ArsC-Independent Reduction to As(III)

To determine As speciation, our assay requires the addition of phosphate to block As(V) uptake and prevent its detection. This approach could be impractical in natural waters with elevated phosphate concentrations (>100 μM). Moreover, Pi addition increases ionic strength and may change the way As interacts with natural ligands, thus altering its bioavailability. In an effort to alleviate the need to amend the sample with inorganic phosphate, we explored the option of taking a genetic approach in preventing the detection of As(V). Previous studies have suggested that As(V) first requires enzymatic reduction to As(III) by ArsC before it can be detected by biosensors (Stocker et al., 2003; Sharma et al., 2013). Indeed, affinity of arsenic to ArsR has only been reported for As(III) (Wu and Rosen, 1993; Shen et al., 2013) and not As(V).

**FIGURE 4:**
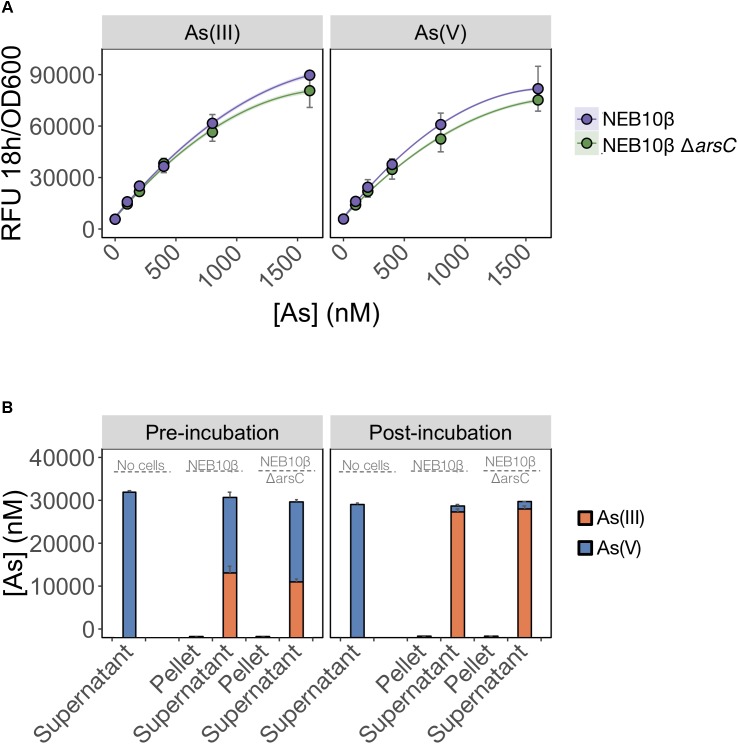
As(V) detection involves an ArsC-independent reduction to As(III). **(A)** As(III) and As(V) detection by the pMP01 biosensor plasmid was compared for the *E. coli* host strain (NEB10-beta) and a deletion mutant lacking arsenate specific reductase (NEB10-beta Δ*arsC*). Data points represent the mean output signal after 18 h of arsenic exposure in MGP of three independently grown cultures, quantified in triplicate. Error bars indicate standard deviations. We found no significant difference between wild-type and mutant strains (*p* = 0.07). **(B)** Speciation analysis following addition of 32 μM As(V) to MGP exposure media (no cells), NEB10-beta cultures and NEB10-beta Δ*arsC* cultures. Treatments were subsampled, fractionated by centrifugation and analyzed before (left panel) and after (right panel) a 3-h incubation. As(III) and As(V) concentrations in pellet and supernatant fractions were quantified using HPLC-ICP-MS. The mean and standard deviation of three independent treatments are presented.

We first tested whether ArsC was solely required for As(V) detection and deleted *arsC* from the biosensor host genome. We predicted that As(V) detection would be limited by the inability of the mutant to reduce As(V) to As(III). We found that deletion of *arsC* had no significant effect on the output signal following exposure to either inorganic arsenic species (**Figure 4A**), indicating that ArsC is not required for As(V) detection.

We then tested the possibility that an ArsC-independent mechanism mediates As(V) reduction in the exposure medium (**Figure 4B**). We compared As(V) reduction efficiency of a wild-type strain harboring the full *ars*-operon to that of an *arsC* mutant. Both the wild-type and Δ*arsC* strains reduced >94% of the As(V) initially provided, with most of the arsenic located in the supernatant fractions. We suspect that the time elapsed between sample collection and speciation analysis by HPLC-ICP-MS is responsible for the reduction observed in the pre-incubation samples. However, the similar reduction rates between wild-type (NEB10-beta) and mutant (Δ*arsC*) treatments post-incubation, strongly support the existence of ArsC-independent As(V) reduction mechanism(s) in *E. coli*. We confirmed these findings by transforming the Δ*arsC* mutant strain from the Keio collection (JW3470) with the biosensor construct (pMP01). The Δ*arsC* JW3470 biosensor responded to As(V) at a similar rate and signal intensity as the exposure to As(III) (data not shown). Furthermore, our mass balance and ICP-MS measurements showed that >94% of As(V) was reduced to As(III), while no As(V) was reduced in our abiotic controls. Although we did not specifically test for extracellular As(V) reduction, to the best of our knowledge the only As(V) reduction pathways in *E. coli* reported in the literature are intracellular. Therefore, our data suggest that >94% of As(V) was available to the *E. coli* cells under these conditions.

Together, these findings suggest that As(V) detection by the *E. coli* biosensor involves a biologically mediated As(V) reduction step, independent of ArsC. Interestingly, a recent study (Chrysostomou et al., 2015) identified an ArsC-independent reduction pathway when screening an *E. coli* Δ*arsC* mutant for resistance to As(V). The authors found that overexpression of Glutathione-*S*-Transferase (GstB) increased As(V) resistance by reducing As(V) to As(III). Our data suggest that intracellular As(V) in *E. coli* is susceptible to non-specific reduction pathways (possibly via Glutathione-*S*-Transferase) in addition to the specific arsenic-inducible pathway (*arsC*). Because GstB is likely essential to the cell central metabolism, a GstB deletion mutant does not currently represent a good candidate as a biosensor host.

### As(III) Uptake Is Affected by Central Carbon Metabolism

The apparent sensitivity of As(V) uptake to media constituents led us to also revisit how As(III) uptake is affected by such media constituents. Previous studies have suggested that 90% of As(III) enters *E. coli* cells via glycerol uptake facilitator channels (GlpF) (Sanders et al., 1997; Rosen and Tamás, 2010). These channels are responsible for the passive transport of water and small hydrophilic molecules including glycerol across the inner membrane (Hénin et al., 2008). Transcriptional induction of *glpF* during growth on glycerol is mediated by the interaction between glycerol-3-phosphate and the GlpR repressor (Lin, 1976; Weissenborn et al., 1992). Expression of *glpF* is repressed by GlpR and sensitive to catabolite repression during growth on preferred carbon sources such as glucose (Weissenborn et al., 1992). We hypothesized that uptake of As(III) is affected by conditions that alter the expression of the *glpF* transporter.

**FIGURE 5:**
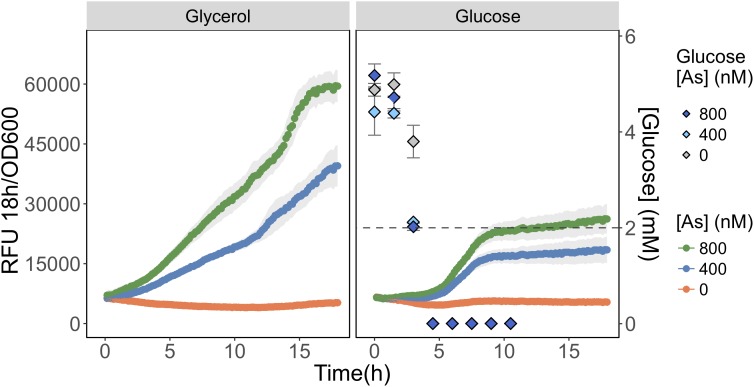
Detection of As(III) is hampered in glucose grown biosensor cultures. As(III) was detected in biosensor cultures grown and exposed in media containing glycerol **(left)** or glucose **(right)** as primary carbon sources. The RFU per OD_600_ is given for the 18 h following arsenic exposure. The concentration of glucose in culture supernatant fractions was quantified using HPLC-ELSD over time and is presented on the secondary *y*-axis. The mean and standard deviations of biological triplicate exposures are represented for both RFU and the glucose concentration estimates. HPLC-ELSD limit of detection is presented as the dotted line.

We compared As(III) detection during growth on a carbon source favoring *glpF* induction (glycerol) to one favoring *glpF* repression (glucose). We observed that fluorescence produced in response to As(III) exposure was twofold higher during growth on glycerol than on glucose, consistent with greater uptake of As(III) in cells growing on glycerol (**Figure 5**). Analysis of glucose consumption in the cultures indicated that the increased signal output occurred after most of the glucose (>60%) was consumed. As a control, glycerol treated cells were also analyzed for glucose content and no glucose was found in any of the triplicate glycerol treatments at *T* = 0 h (data not shown). This result is consistent with catabolite repression of the *glpF* transporter leading to reduced As(III) uptake during exponential growth on glucose, with increased detection occurring under conditions following relaxation of catabolite repression.

### Application of the Biosensor to Arsenic Quantification in Lake Samples

We were interested in testing the performance of the biosensor in quantifying total arsenic levels in environmental samples. We chose Yellowknife, Northwest Territories in subarctic Canada as our study area. Gold mine operations in this area have severely affected the surrounding lakes due to the atmospheric deposition of arsenic trioxide (As_2_O_3_) from Giant Mine roaster stack emissions (Galloway et al., 2018). The heterogeneity of the underlying bedrock (Villeneuve and Relf, 1998; Yamashita and Creaser, 1999) has resulted in lakes with diverse chemical profiles. Despite cessation of goldmining operations in 2004, legacy arsenic contamination in many lakes still far exceeds Canadian guidelines for the protection of aquatic life (5 μg/L or 67 nM) and drinking water standards (10 μg/L or 133 nM).

**FIGURE 6:**
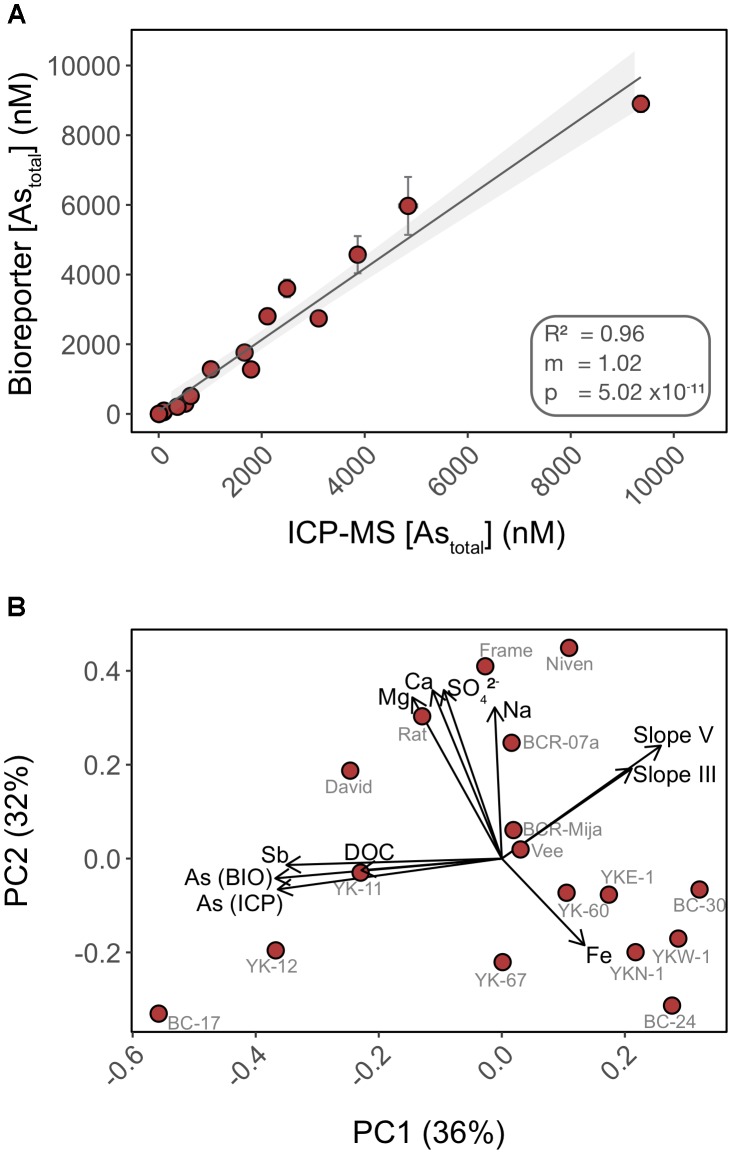
Total inorganic arsenic concentrations are accurately quantified using the biosensor assay through a wide range of water chemistry profiles. **(A)** Cross-analysis of arsenic quantification in 17 surface water samples using the biosensor and HPLC-ICP-MS. **(B)** PCA of the surface water chemistry of 17 lakes in Yellowknife. Each PCA point represents an individual lake separated by Euclidian distances as a metric of variations in water chemistry profile. Overlaid are biplot vectors, pointing in the direction of most rapid change in a specified environmental variable. The strength of the gradient direction is presented by the length of the vector. Standard addition slopes for As(III) and As(V) are used as proxies for their respective bioavailability.

We compared the detection of total inorganic arsenic (As_total_) by the biosensor to that of HPLC-ICP-MS using water samples collected from 17 lakes in the area surrounding Yellowknife (**Figure 6A**). Speciation analysis by HPLC-ICP-MS indicated that >99% of the inorganic arsenic in the lake surface water samples was in the form of As(V) (data not shown), and <1% was in the form of As(III), arsenobetaine or organoarsenic species. This result was expected as we did not chemically preserve the samples (see “Materials and Methods” section for details). The biosensor accurately quantified total inorganic arsenic (*R*^2^ = 0.96) over the entire range of arsenic concentrations in the lake samples (**Figure 6A**). The high accuracy of As(V) quantification by the biosensor is likely due to the improved detection of As(V) in the non-selective exposure medium, which we have found likely permits uptake of greater than 94% of As(V) into the biosensor host (**Figure 4B**). Thus, our data suggest that most of the legacy inorganic arsenic from anthropogenic sources in these environmental samples is available for microbial uptake.

In addition to quantifying legacy arsenic contamination, we also assessed bioavailability of newly added As(III) and As(V) to natural water samples. We performed standard additions of both As(III) and As(V) in 17 surface lake water samples of contrasting water chemistry (**Supplementary Figure S2**). The fluorescence response of the biosensor to standard additions varied with lakes [slopes ranged from -0.46 to 146.16 for As(V) and 24.64 to 170.05 for As(III)] when compared to the calibration curve performed in exposure media, suggesting that water chemistry affected the ability of the biosensor to detect newly added As(III) and As(V) (e.g., BC-17, YK67). Furthermore, and surprisingly, a subset of lakes exhibited a muted response to newly added As(III) and As(V) when compared to controls (e.g., BC-17, YK-12, YK67, Vee). This muted response could be indicative of a lower bioavailability of the newly added Arsenic species, compared to “older” legacy arsenic already present in the system. This matrix effect is likely a consequence of direct or indirect interactions between arsenic and chemical constituents of the water samples.

We ran a principal component analysis (PCA) to explore which chemical variables may contribute to explain the variation observed in arsenic bioavailability between lakes (**Figure 6B**) (Oksanen et al., 2007). Biplot vectors include standard addition slopes for As(III) and As(V) as proxies for their respective bioavailability along with lake chemical variables. Principal components 1 and 2 explained a total of 68% of the variance. The absence of clustering between lakes is indicative of heterogeneity in water chemistry profiles. The vectors corresponding to arsenic bioavailability (slopes) were orthogonal to vectors for the following chemical constituents (Fe, Na, Ca, and Mg) suggesting that these matrix components have little control on the bioavailability of added arsenic in these samples, supporting our experimental data (**Figure 2**). In contrast, the slope vectors had closer alignment, in opposite directions, with DOC (dissolved organic carbon), antimony (Sb) and legacy arsenic concentration [As (ICP) and As (BIO)] vectors. We found a significant negative correlation between As(III) bioavailability and [DOC] (*R*^2^ = 0.212; *p* = 0.04) but not for As(V) (*R*^2^ = 0.09; *p* = 0.13) (**Supplementary Figure S3**). Together these data suggest differential bioavailability of new vs. old (legacy) arsenic that might be explained by interactions with matrix constituents such as DOC. DOC has been shown to form ternary complexes with As(III) and As(V) (Lenoble et al., 2015) and to control arsenic distribution in the sediments of lakes in this region (Galloway et al., 2018). The role of DOC on arsenic bioavailability remains to be tested.

## Conclusion

Whereas biosensors have routinely detected As(III) at environmentally relevant concentrations, detection of As(V) has been less reliable. We found that elimination of inorganic phosphate from the exposure medium, led to an improved detection of As(V). The selective inclusion of phosphate in the exposure media proved to be a useful tool for estimating arsenic speciation under controlled laboratory conditions (**Figures 1**, **3A**) even when the cells are faced with a high background of As(V) (**Figure 3B**). The mechanism responsible for the inhibition of As(V) uptake at high Pi concentrations likely involves competition between Pi and As(V) at the Pit transport site, or to the reduced expression of the high-affinity phosphate transporter system (Pst). Contrary to previous suggestions, we were surprised to find the existence of an ArsC-independent mechanism of As(V) reduction relevant for As biosensor applications (**Figure 4**). We also saw that As(III) detection (**Figure 5**) is affected by the carbon source used in the bioassay. These findings have implications for environments where available carbon sources might alter As(III) bioavailability and thus its biotransformation by microbial communities. Moreover, the pervasiveness of non-specific As(V) reduction in the environment has never been investigated, and, as previously described for mercury (Grégoire and Poulain, 2016), could act as a major route for environmental arsenic redox cycling.

Our field work tested the impacts of heterogeneous environments on the detection of legacy arsenic contamination (**Figure 6A**). Our biosensor assay data were in agreement with HPLC-ICP-MS data. Despite the accurate quantification of legacy arsenic contamination, we observed a muted response to newly added As species in several water samples (**Supplementary Figure S2**). We identified naturally occurring dissolved organic carbon as a potential contender in the environmental control of As bioavailability. We propose that DOM has the potential to kinetically control the bioavailability of arsenic; further work is warranted to characterize the nature of the As-DOM interactions and better predict As mobility and availability in the environment.

## Author Contributions

MP, AH, and AP planned the experiments. MP and AH carried out the experiments, designed the biosensor exposure assay, and analyzed the data. MP wrote the manuscript with support from AH and AP. AP supervised the project.

## Conflict of Interest Statement

The authors declare that the research was conducted in the absence of any commercial or financial relationships that could be construed as a potential conflict of interest.
