# Supplementary material for: Insights Into Arsenite and Arsenate Uptake Pathways Using a Whole Cell Biosensor
Source: Front Microbiol. 2018 Oct 2;9:2310. doi: 10.3389/fmicb.2018.02310 (PMC6176005; doi:10.3389/fmicb.2018.02310)
Supplement: Supplementary file 1 [file Data_Sheet_1.PDF]

Supplementary Material

Insights into Arsenite and Arsenate Uptake Pathways Using a Whole Cell Biosensor

Martin P. Pothier[1], Aaron Hinz[1], Alexandre Poulain[1*]

*** Correspondence:** Dr. Alexandre Poulain: apoulain@uottawa.ca

1 Supplementary Table

1.1 Supplemental Table 1: Media recipe and constituents concentration

Reagents	Constituents	[Constituents] in stock solution (M)	[Constituents] in MGP growth media (M)	[Constituents] in MGP exp media (M)	[Constituents] in MIP exp media (M)
MOPS buffer (pH=7.150)	$C_7H_{14}NO_4S$	0.40	0.020	0.020	0.020
	NaCl	0.40	0.020	0.020	0.020
	KCl	0.40	0.020	0.020	0.020
	$(NH_4)_2SO_4$	0.64	0.032	0.032	0.032
β-glycerophosphate	$(HOCH_2)_2CHOP(O)(ONa)_2 \cdot xH_2O$	0.40	0.001	0.001	0.001
Magnesium sulphate	$MgSO_4$	2.0	$1.25x10^{-3}$	$2.00x10^{-5}$	$2.00x10^{-5}$
Glycerol	$C_3H_8O_3$	4.0	0.030	0.005	0.005
Amino acids	L-Leucine	0.075	$2.28x10^{-4}$	$2.28x10^{-4}$	$2.28x10^{-4}$
	L-Isoleucine	0.075	$2.28x10^{-4}$	$2.28x10^{-4}$	$2.28x10^{-4}$
	Valine	0.075	$2.28x10^{-4}$	$2.28x10^{-4}$	$2.28x10^{-4}$
Trace elements 1 (0.1M H_2SO_4)	$NiCl_2 \cdot 6H_2O$	$8.41x10^{-5}$	$8.41x10^{-8}$	—	—
	$CoCl_2 \cdot 6H_2O$	$4.20x10^{-5}$	$4.20x10^{-8}$	—	—
	$ZnSO_4$	$3.48x10^{-4}$	$3.48x10^{-7}$	—	—
	$MnSO_4$	$9.41x10^{-3}$	$9.41x10^{-6}$	—	—
Trace elements 2 (0.1M NaOH)	H_3BO_3	$4.85x10^{-3}$	$4.85x10^{-6}$	—	—
	$Na_2MoO_4 \cdot 2H_2O$	$1.24x10^{-4}$	$1.24x10^{-7}$	—	—
Pi buffer (pH=7.150)	$NaH_2PO_4 \cdot H_2O$	0.43	—	—	0.01
	K_2PO_4	0.57			

2 Supplementary Figures

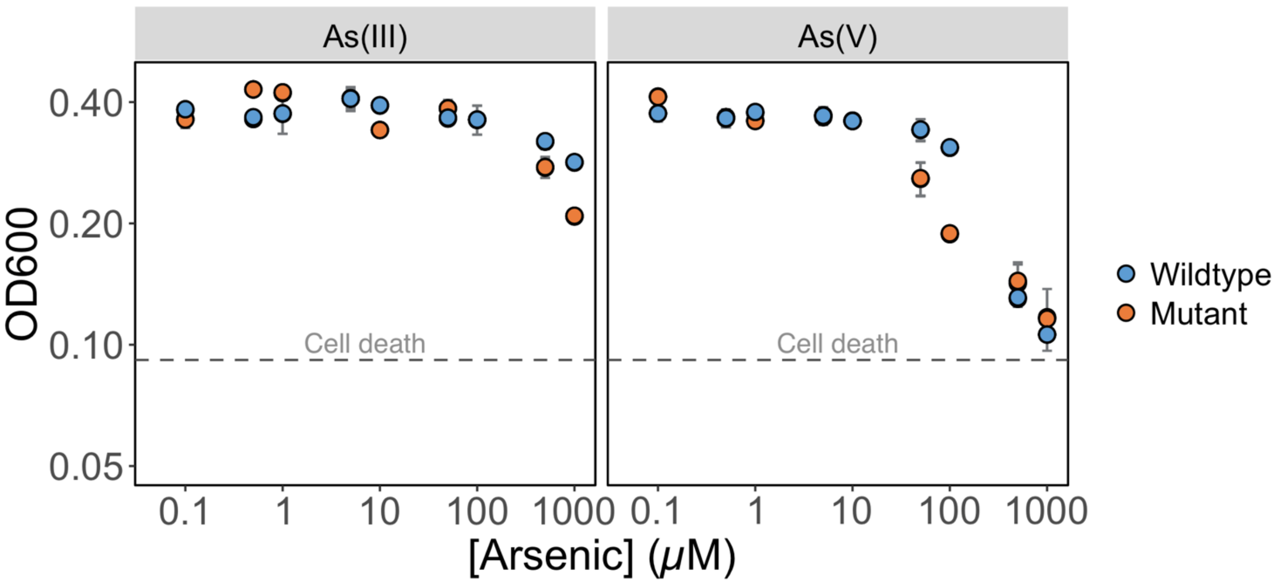

Supplementary Figure 1. Δ*arsC* deletion mutants are more sensitive to As(V) than wild-type *E. coli* NEB10-beta. Minimal inhibitory concentrations of As(III) (left panel) and As(V) (right panel) were determined for both wild-type and mutant strains using standard As exposure protocol in MGP exposure medium. Cell culture yields at 18 hours are presented on the Y-axis. Error bars represent the standard deviation of biological triplicates. For As(III), toxicity was not observed below 500 μM for both strains. For As(V), a decrease in yield was observed at 100 μM for wildtype, but 50 μM for Δ*arsC* deletion mutant. The dashed lines represent complete inhibition of cell growth.

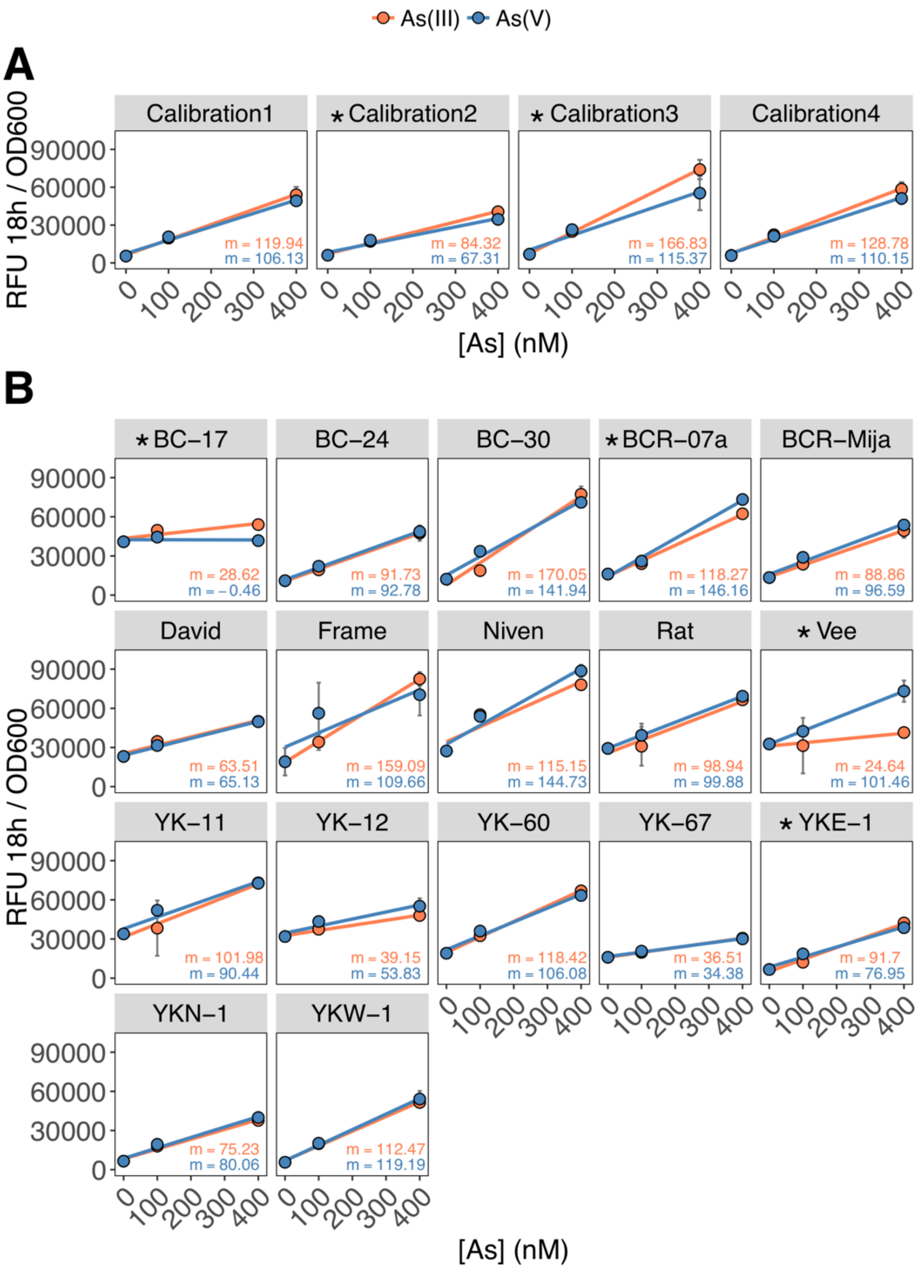

Supplementary Figure 2. Bioavailability of arsenic is sensitive to sample matrix. Selected lakes were located in close proximity to Yellowknife, Northwest Territories in subarctic Canada. Regression lines were fitted to standard additions of As(III) (orange) and As(V) (blue) using **(A)** ultrapure water (calibration, performed daily), and **(B)** natural surface waters from 17 lakes of varying water chemistry profiles. Input As concentration is denoted on the X-axis in nanomolar. Output signal (RFU) on the Y-axis is normalized to culture yield (OD600) after 18 hours of growth. Lake names are found in the panel title. Regression line slopes for As(III) is indicated in orange colored text and As(V) in blue colored text at the bottom right of each panel. Regression intercepts vary among lakes due to the naturally occurring As concentrations in each lake. Differences in slopes between lakes is used as a proxy for a change in bioavailability of newly added arsenic species.

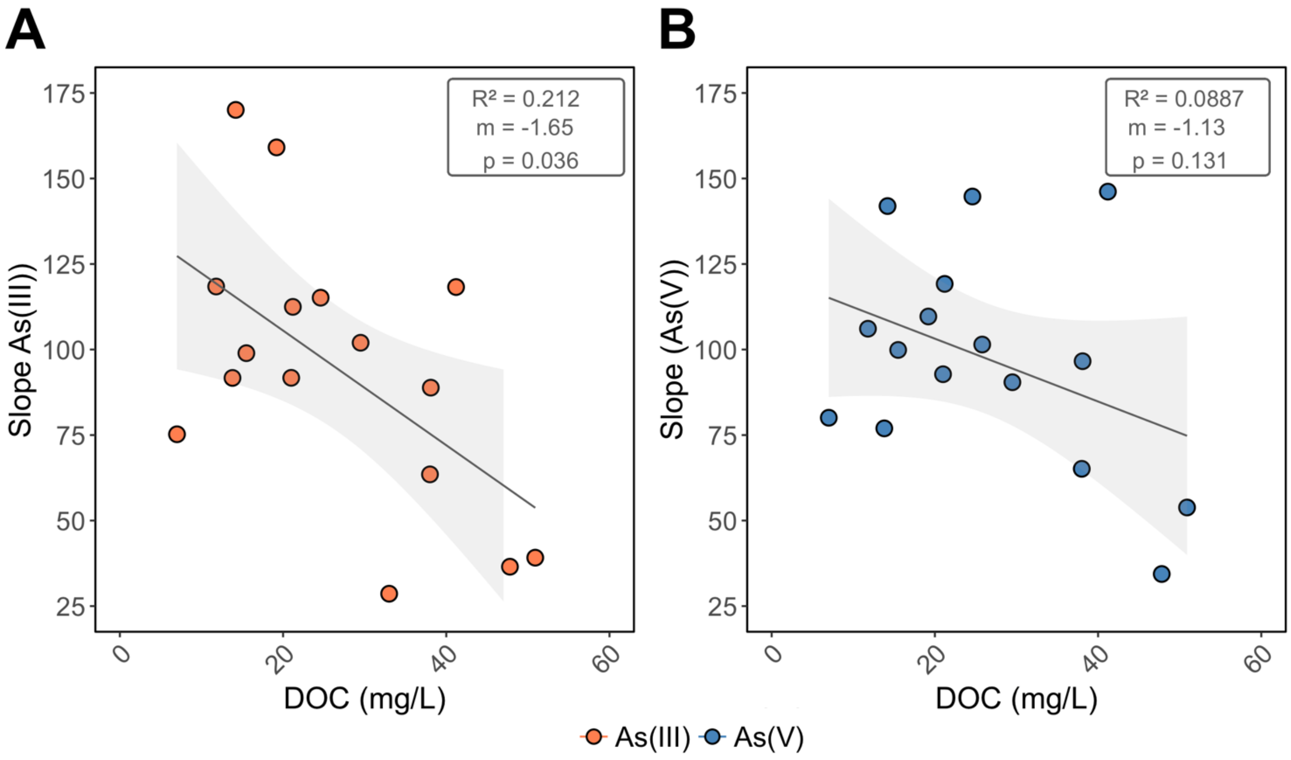

Supplementary Figure 3. Relationship between [DOC] on As(III) and As(V) bioavailability. Dissolved organic carbon (DOC) concentrations in each lake are plotted on the X-axis. As a proxy for arsenic bioavailability, slope data gathered from arsenic standard additions of supplemental Figure 2 is presented on the Y-axis. Significant negative correlation is observed between As(III) bioavailability and [DOC].

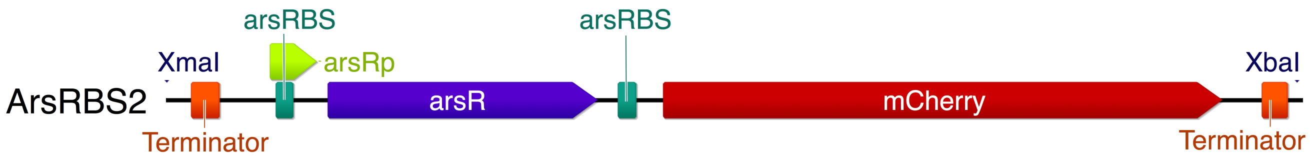

Supplementary Figure 4. Map representing the construct inspired by Stocker et al. 2003.
